# Awareness and Knowledge of Common ENT-Related Issues Among the Population in the Qassim Region: A Cross-Sectional Study

**DOI:** 10.7759/cureus.71785

**Published:** 2024-10-18

**Authors:** Mazyad Alenezi, Faisal A Al-Harbi, Razan S Alharbi, Abdullah S Alayed, Emad K AlOadah, Faisal Almutairi, Munthir M Alanazi

**Affiliations:** 1 Department of Otolaryngology - Head and Neck Surgery, Qassim University, Buraydah, SAU; 2 College of Medicine, Qassim University, Buraydah, SAU

**Keywords:** awareness, ent disorders, knowledge, public health, qassim region, saudi arabia

## Abstract

Background

ENT disorders are common, leading many people to seek medical advice. A common misconception among the population is that upper respiratory tract viral infections can be treated with antibiotics, despite their ineffectiveness against viruses. Assessing the level of knowledge and awareness regarding common ENT-related issues among the population in the Qassim region is therefore crucial.

Methods

We conducted a cross-sectional study using a random sampling technique to survey 415 participants aged 18 and above. Data were collected through an online self-administered questionnaire and analyzed using SPSS version 26 (IBM Corp., Armonk, NY, USA). The questionnaire assessed participants' knowledge of various ENT issues, and scores were calculated to gauge overall awareness.

Results

The majority of participants demonstrated a satisfactory level of knowledge, with a mean knowledge score of 9.37 ± 3.59. Significant gender differences were observed, with females displaying higher knowledge levels (61.9%, p < 0.05). Additionally, higher educational attainment was positively correlated with better awareness of ENT issues.

Conclusion

While general awareness of ENT-related issues in the Qassim region is adequate, specific knowledge gaps remain, particularly regarding the treatment of viral infections. Targeted educational initiatives are needed to address these gaps, focusing on groups with lower awareness levels.

## Introduction

The term "ear, nose, and throat" (ENT) disorders, also referred to as "otolaryngology disorders," refers to a group of diseases that affect the ear, nose, and throat [1]. According to a study, ENT disorders are among the most common reasons that patients visit emergency departments (ED). Patients are regularly referred to ENT specialists by general practitioners (GPs) and other medical specialists. About 10% of all primary care cases that present have ENT symptoms [2].

Since ENT disorders can lead to morbidities that impact bodily functions such as taste, smell, speaking, breathing, swallowing, voice production, lower respiratory tract protection, hearing, and mucus clearance, knowledge of ENT disorders is very important [3]. The three main ENT issues are head and neck cancer, rhinosinusitis, and hearing loss, according to published research [4,5].

A cross-sectional study conducted in Nigeria looked at 290 respondents' knowledge, attitudes, and awareness of using cotton buds. A total of 278 of the 290 participants' responses, whose ages ranged from 18 to 65, were examined. Forty-three percent of the respondents were civil servants, and the majority (72.3%) had postsecondary education. The majority of participants (92.8%) said they cleaned their ears with cotton buds, mostly because their ears itched (57.8%). Only 44.9% of respondents were aware that cotton buds could harm ears, indicating a low level of awareness of this danger. In contrast, 61.2% of respondents said that using cotton buds for ear care was beneficial. The vast majority (74.1%) had not been informed about the risks associated with cleaning one's ears with cotton buds [6].

In Makkah, Saudi Arabia, a cross-sectional study looked into students' attitudes and awareness of prevalent ENT issues. There were 1080 respondents from Makkah City in the survey. Individuals who were over the age of 20, female, and held a bachelor's or university degree showed a considerably higher level of awareness of prevalent ENT illnesses [7].

Researchers evaluated students at Saudi Arabia's Najran University and their understanding and practices about prevalent ENT issues in a cross-sectional study. The participants were primarily female (69.0%), over the age of twenty-one (84.1%), and enrolled in health colleges (45.2%). According to the results, 37.8% of respondents knew a good deal about ENT concerns, while 62.2% didn't. The majority of respondents (87.2%) said that acute ENT issues should be treated in a hospital. Nationality and clinical history did not indicate any significant relationships with knowledge levels about ENT disorders, but age, gender, health college affiliation, college, and department did [8].

A cross-sectional study evaluated public practices and knowledge levels on prevalent ENT-related issues in the Northern Borders Region of Saudi Arabia. Of the 363 participants in the study, 248 (68.3%) had a medical background, and the majority (n = 326, 89.8%) were over the age of 20 years. The results showed that 224 individuals (61.8%) had an inadequate understanding of ENT-related issues, while 139 participants (38.2%) showed high knowledge. Knowledge levels were shown to be significantly correlated with age, gender, and educational attainment. Nonetheless, in this particular context, there were no statistically significant correlations found between knowledge levels and marital status, place of residence, occupation, or medical background [9].

The purpose of this study was to assess the level of awareness and knowledge of common ENT-related issues among the population of the Qassim region, Saudi Arabia.

## Materials and methods

Study design, setting and time frame

A cross-sectional study was done in the Qassim region of Saudi Arabia from June to August 2024. Qassim region is one of the 13 provinces of Saudi Arabia. Located at the heart of the country near the geographic center of the Arabian Peninsula, it has a population of 1,336,179 and an area of 58,046 km^2^.

Study participants

The inclusion criteria were residents of the Qassim region of Saudi Arabia aged 18 years and above. The exclusion criteria were residents less than 18 years old, residents outside the Qassim region, and those who refused sharing in the study.

Sample size

The Raosoft online calculator website was used for the calculation of the sample size. With the use of a 5% margin of error, 95% confidence interval and a population of Al-Qassim region of 1,336,179 the minimum sample size needed was 385 participants [10].

Data collection

The survey was conducted using an electronic questionnaire written in Arabic. Data was collected through an online self-administered questionnaire that was used in previous studies [9,11]. The questionnaire was distributed to the participants through social media platforms. The participants were asked to fill the questionnaire after they received a complete explanation of the purpose of the study. The questionnaire included three primary sections:

1. Demographic information (age, gender, marital status, level of education, and occupation).

2. General Knowledge about cotton buds, newborns’ hearing screening, upper respiratory tract viral infections treatment, influenza vaccine, vitamin C, using olive oil as ear drops, and if dizziness and vertigo have the same meaning.

3. Ear and hearing diseases and nose, throat, and laryngeal diseases.

Participants’ knowledge was classified as “excellent” if they obtained a ≥75% score, “good” if they had a score between 74% and 50%, and “poor” if their score was <50%. Acceptable knowledge was defined as obtaining a knowledge score ≥50% (9).

Ethical considerations

An ethical approval for the study was obtained from the Institutional Review Board (IRB) of the Regional Research Ethics Committee, Qassim Province, Saudi Arabia.

Data analysis

Data were statistically analyzed using SPSS version 26 (IBM Corp., Armonk, NY, USA). To investigate the association between the variables, the Chi-squared test (χ2) was applied to qualitative data that was expressed as numbers and percentages. Quantitative variables were expressed as mean and standard deviation (Mean ± SD) and a p-value of less than 0.05 was considered statistically significant.

## Results

Of the studied 415 participants, 181 (43.6%) had an age ranging from 18-30 years, 257 (61.9%) were females, and 228 (54.9%) were married. Of them, 193 (46.5%) had a bachelor’s degree in education, and 130 (31.3%) were unemployed (Table 1).

**Table 1:** Distribution of the studied participants according to their demographic characteristics (No.: 415).

Variable	No. (%)
Age (years)	
18-30	181 (43.6)
31-50	165 (39.8)
>50	69 (16.6)
Gender	
Female	257 (61.9)
Male	158 (38.1)
Marital status	
Widow	7 (1.7)
Single	169 (40.7)
Married	228 (54.9)
Divorced	11 (2.7)
Educational level	
Less than secondary school	8 (1.9)
Secondary school	42 (10.1)
Diploma	58 (14)
University student	91 (21.9)
Bachelor’s degree	193 (46.5)
Postgraduate	23 (5.5)
Occupation	
I work in the medical sector	9 (2.2)
I work in other sectors	200 (48.2)
Student in the medical sector	39 (9.4)
Student in other sectors	37 (8.9)
I do not work	130 (31.3)

Table 2 illustrates the participants' knowledge about common ENT-related issues and ear and hearing diseases. It was found that most of the participants (288, 69.4%) disagreed that cotton buds are the safest way for cleaning your ears, 250 (60.2%) agreed that newborns’ hearing can be screened and 125 (30.1%) disagreed that upper respiratory tract viral infections can be treated with antibiotics. The majority (254, 61.2%) agreed that it is recommended to take the influenza vaccine every year, but only 120 (28.9%) disagreed that influenza vaccine is not recommended for diabetic and hypertensive patients. Only 65 (15.7%) of them disagreed that vitamin C can prevent and cure the common cold. Almost half (203, 48%) disagreed that using olive oil as ear drops can cure ear diseases and the majority (259, 62.4%) disagreed that dizziness and vertigo are the same. As for knowledge about ear and hearing diseases, 368 (88.7%) and 324 (78.1%) participants agreed that hearing impairment in children can cause attention deficit and affect school performance, and may affect social life respectively. Of them, 274 (66%) agreed that continuous noise exposure can harm your hearing and may cause hearing loss. As for hearing aids, 325 (78.3%) agreed that they are one of the most common ways to improve hearing loss in elderly patients. The majority (371, 89.1%) agreed that inner ear infections can cause vertigo, but only 93 (22.4%) agreed that hearing aids can be used in children <12 months. More than half (213, 51.3%) disagreed that all ear pain is necessarily middle‑ear infections and 360 (86.7%) agreed that sudden hearing loss is an emergency condition and needs an immediate medical assessment.

**Table 2:** Participants’ responses on knowledge questions related to common ENT-related issues and ear and hearing diseases (No.: 415).

Variable	False	True	I don’t know
	No. (%)	No. (%)	No. (%)
General knowledge			
Cotton buds are the safest way for cleaning your ears. (false)	288 (69.4)	93 (22.4)	34 (8.2)
Newborns’ hearing can be screened. (true)	58 (14)	250 (60.2)	107 (25.8)
Upper respiratory tract viral infections can be treated with antibiotics. (false)	125 (30.1)	150 (36.1)	140 (33.7)
It is recommended to take influenza vaccine every year. (true)	76 (18.3)	254 (61.2)	85 (20.5)
Influenza vaccine is not recommended for diabetic and hypertensive patients. (false)	120 (28.9)	112 (27)	183 (44.1)
Vitamin C can prevent and cure common cold. (false)	65 (15.7)	299 (72)	51 (12.3)
Using olive oil as ear drops can cure ear diseases. (false)	203 (48)	101 (24.3)	111 (26.7)
Dizziness and vertigo are the same. (false)	259 (62.4)	97 (23.4)	59 (14.2)
Ear and hearing diseases			
Hearing impairment in children can cause attention deficit and affect school performance. (true)	17 (4.1)	368 (88.7)	30 (7.2)
Hearing loss may affect social life. (true)	68 (16.4)	324 (78.1)	23 (5.5)
Continuous noise exposure can harm your hearing and may cause hearing loss. (true)	58 (14)	274 (66)	83 (20)
Hearing aids are one of the most common ways to improve hearing loss in elderly patients. (true)	25 (6)	325 (78.3)	65 (15.7)
Inner ear infections can cause vertigo. (true)	10 (2.4)	371 (89.1)	34 (8.2)
Hearing aids can be used in children <12 months. (true)	105 (25.3)	93 (22.4)	217 (52.3)
All ear pain is necessarily middle‑ear infections. (false)	213 (51.3)	60 (14.5)	142 (34.2)
Sudden hearing loss is an emergency condition and needs an immediate medical assessment. (true)	15 (3.6)	360 (86.7)	40 (9.6)

**Figure 1:**
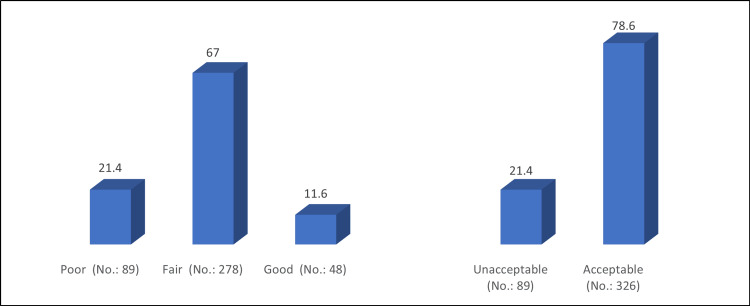
Percentage distribution of participants’ level of knowledge about common ENT-related issues, and ear and hearing diseases (No.: 415).

The mean knowledge score was 9.37 ± 3.59. Based on the knowledge score classification, 326 (78.6%) of the participants had an acceptable knowledge level, while 89 (21.4%) had an unacceptable knowledge level (Figure 1).

**Figure 2:**
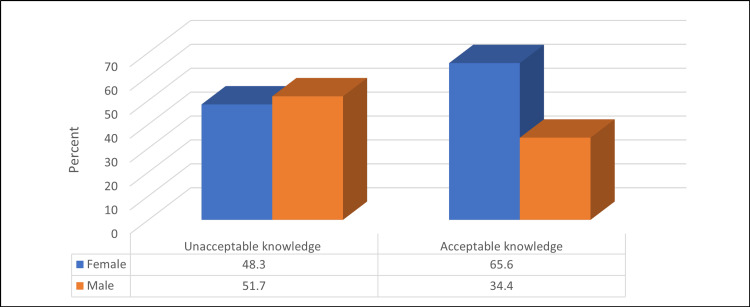
Relationship between level of knowledge about common ENT-related issues and ear and hearing diseases and participants’ gender (No.: 415). N.B.: (χ2 = 8.9, p-value = 0.003)

Table 3 and Figure 2 illustrate that the prevalence of acceptable knowledge level was significantly higher among female participants compared to males, 214 (65.6%) vs. 112 (34.4%) (p ≤ 0.05). On the other hand, a non-significant relationship was found between the level of knowledge and all other participants’ demographics (p > 0.05).

**Table 3:** Relationship between level of knowledge about common ENT-related issues and ear and hearing diseases and participants’ demographics (No.: 415).

Variable	Knowledge level		χ2	p-value
	Unacceptable knowledge No. (%)	Acceptable knowledge No. (%)		
Age (years)				
18-30	42 (47.2)	139 (42.6)	2.4	0.3
31-50	37 (41.6)	128 (39.3)		
>50	10 (11.2)	59 (18.1)		
Gender				
Female	43 (48.3)	214 (65.6)	8.9	0.003
Male	46 (51.7)	112 (34.4)		
Marital status				
Widow	1 (1.1)	6 (1.8)	1.32	0.723
Single	40 (44.9)	129 (39.6)		
Married	45 (50.6)	183 (56.1)		
Divorced	3 (3.4)	8 (2.5)		
Educational level				
Less than secondary school	4 (4.5)	4 (1.2)	4.06	0.407
Secondary school	10 (11.2)	32 (9.8)		
Diploma	12 (13.5)	46 (14.1)		
University student	20 (22.5)	71 (21.8)		
Bachelor’s degree	40 (44.9)	153 (46.9)		
Postgraduate	3 (3.5)	20 (6.1)		
Occupation				
I work in the medical sector	1 (1.1)	8 (2.5)	9.03	0.06
I work in other sectors	47 (52.8)	153 (46.9)		
Student in the medical sector	2 (2.2)	37 (11.3)		
Student in other sectors	6 (6.7)	31 (9.5)		
I do not work	33 (37.1)	97 (29.8)		

## Discussion

This study evaluated the population's awareness and understanding of prevalent ENT-related disorders in the Saudi Arabian province of Qassim.

It was evident from our results that 326 of the participants (78.6%) knew the fundamentals of ENT issues. However, a prior survey conducted in Riyadh discovered that while 18.4% of people had good knowledge, only 2.3% of people had exceptional knowledge. The remainder, or 79.4%, had a limited awareness (<50% score) of illnesses related to the ENT. However, 38.2% of participants in a different survey conducted in Saudi Arabia's Northern Borders Region showed a high level of knowledge of matters pertaining to otorhinolaryngology [9].

Similar to a study conducted in Saudi Arabia's Northern Borders [9], the current study found that women had higher levels of awareness. This finding shows that as women are typically the primary carers, they have greater exposure to health-related information. The study's results are consistent with those of Alassaf et al.'s investigation, which showed that women possessed more knowledge about common issues pertaining to the nose, throat, and ears because they are typically regarded as the primary carers for family health issues rather than men [12]. A prior study conducted at Najran University to assess students' understanding and practices about common ENT disorders revealed similar gender disparity [8]. However, there was no significant correlation discovered between the study participants' degree of knowledge and any of the other demographic variables such as gender. On the other hand, participants in a study conducted in the Makkah region of Saudi Arabia who were older than 20 years had a greater understanding of issues connected to otorhinolaryngology than those who were younger [7] - an outcome that aligns with the 2023 study by Assiri et al. [8].

Interestingly, 254 (61.2%) of participants felt that yearly influenza vaccinations were necessary, which is in line with a 2019 poll conducted in the Qassim region, where 56.9% of respondents concurred [13]. The same conclusion was reached in a different Saudi study, where 78.9% of participants knew well enough about the value of yearly immunization against the flu [9]. While further efforts are needed to increase awareness even further, this consistency suggests that public health programs might be having a positive impact on people.

Regarding ENT safety awareness, 288 (69.4%) of participants understood that cotton buds could pose a risk, and 274 (66%) were aware that exposure to noise can impair hearing. The majority of participants, according to Jalaladdin et al., were well-informed about the possible risks associated with using cotton earbuds [7]. Moreover, a 2013 Milan (Italy) study found that most participants were well aware of the potential risks associated with cotton earbuds [14].

There was a notable disparity in awareness of the use of hearing aids in newborns (93, 22.4%), but a larger percentage of participants (325, 78.3%) were well aware of them and their advantages. These results are in line with earlier research conducted in Riyadh and the Northern Borders region [9,11]. The disparity in awareness highlights the value of tailored education programs to close particular gaps, especially in the treatment of pediatric hearing impairments.

Of the participants, 360 (86.7%) participants agreed that sudden hearing loss was an emergency. This conclusion is consistent with studies conducted in Makkah (93.6%) and Riyadh (96.6%), where the majority of participants had a high level of awareness of what to do in the event of unexpected hearing loss [11, 15]. The results align with those of Alshehri et al. (2019), who noted a high degree of awareness of the best course of action to be followed in the event of an unexpected ear, nose, or throat issue, which is to visit a hospital [5]. The results of the present study were superior to those of an Italian study in this regard, wherein 80% of the participants indicated that they would visit a hospital in the event of an abrupt loss of hearing [14]. This widespread recognition of the value of hearing in culture may be the consequence of effective health education programs emphasising the value of hearing. However, disparities in awareness suggest that there may be differences in the calibre and availability of health information, calling for more investigation.

The percentage of survey participants, who disagreed that vitamin C may both prevent and treat the common cold, was only 15.7%. On the other hand, 64.2% of participants in the Alkhalifah et al.’s study were well-versed in the application of vitamin C for both treating and preventing colds [9]. However, our results conflict with those of a Saudi Arabian study by Jalaladdin et al. that demonstrated a high level of understanding regarding the role that vitamin C and vaccinations play in preventing influenza [7].

A sizable percentage of participants (88.7%) stated that children with hearing loss have poorer academic performance, and 78.1% said that it has an impact on social relations. This result is consistent with that of Alkhalifah et al.’s study, in which the majority of participants had a high level of understanding of the impact of hearing loss on children's social and academic performance (89.8% and 82.1%, respectively) [9].

Nevertheless, just 60.2% of respondents were aware of neonatal hearing screenings, which is in line with the Makkah study [15]. Nonetheless, prior studies conducted in Saudi Arabia's northern border region revealed that 70% of participants had good knowledge of the significance of newborn test hearing. This discrepancy suggests that in order to prevent developmental delays and improve quality of life, additional public health initiatives supporting early hearing examinations are needed.

This study and others of a similar nature help the public health system by pointing out areas of weakness that require attention in order to make the system better. Additionally, it provides a thorough review of public awareness to specialized physicians, assisting them in dispelling myths during patient visits.

The inclusion of a question in the demographic section that differentiates between participants who work or study in the medical sector and those who do not is a noteworthy strength of this study. This methodology enables a more focused evaluation of public knowledge and awareness than other research that might not have excluded students or medical professionals.

Limitations

The use of an online questionnaire may have led to some participants misunderstanding or misinterpreting questions, as there was no specialized person available to provide clarification in real-time. This could have resulted in inaccurate responses. Additionally, individuals without internet access or those who are uncomfortable with online surveys were unable to participate, potentially limiting the study's reach.

## Conclusions

This study highlights the generally adequate knowledge of common ENT-related issues among residents of the Qassim region, with notable exceptions in understanding specific aspects of ear health and vaccine recommendations. The observed gender and education disparities in knowledge levels suggest that targeted and inclusive educational interventions could bridge these gaps. Enhancing public health education, particularly among males and individuals with lower educational attainment, is essential for improving overall ENT health awareness and outcomes in the region.
